# Efficacy and Tolerability of Lopinavir/Ritonavir- and Efavirenz-Based Initial Antiretroviral Therapy in HIV-1-Infected Patients in a Tertiary Care Hospital in Beijing, China

**DOI:** 10.3389/fphar.2019.01472

**Published:** 2019-12-12

**Authors:** Bin Su, Yin Wang, Ruifeng Zhou, Taiyi Jiang, Hongwei Zhang, Zaicun Li, An Liu, Ying Shao, Wei Hua, Tong Zhang, Hao Wu, Shenghua He, Lili Dai, Lijun Sun

**Affiliations:** ^1^Center for Infectious Diseases, Beijing Youan Hospital, Capital Medical University, Beijing, China; ^2^Beijing Key Laboratory for HIV/AIDS Research, Beijing, China; ^3^Center for Infectious Diseases, Public Health Clinic Center of Chengdu, Chengdu, China

**Keywords:** human immunodeficiency virus, first-line therapy, antiretroviral therapy, lopinavir/ritonavir, efavirenz, adverse effects

## Abstract

**Background:** Lopinavir/ritonavir (LPV/r) is a major antiretroviral treatment in China, but little is known about the performance of first-line LPV/r-based regimen in treatment-naïve patients with human immunodeficiency virus type 1 (HIV-1) infection. This study aims to assess the efficacy and adverse effect events of LPV/r plus lamivudine and tenofovir or zidovudine as an initial antiretroviral treatment in HIV-1-infected individuals for whom cannot take efavirenz (EFV) or is allergic to EFV.

**Methods:** We performed a retrospective study of patients registering with the China’s National Free Antiretroviral Treatment Program from July 2012 to January 2017, followed at a tertiary care hospital in Beijing, China. The primary outcome was the proportion of subjects with HIV-1 RNA ≤40 copies/ml at 6 and 24 months of treatment. We assessed the immunological response and adverse events.

**Results:** In total, 4,862 patients were enrolled in the study and 237 were eligible for analysis in each study arm. During the first six months, virological suppression was better with the LPV/r-based regimen than with the EFV-based regimen (93.80 vs 87.80% for *P* < 0.05). Viral suppression rates continued to increase until 12 months, remain steady thereafter until 24 months, for both groups. The multilevel analysis revealed that patients in the LPV/r group were more likely to display improvements in CD4 T-cell count over time than those in the EFV group (*P* < 0.001). Grade 3 or 4 laboratory adverse events were observed in 14 patients (5.91%) from the LPV/r group and three patients (1.20%) in EFV group.

**Conclusion:** Our findings demonstrate that LPV/r-containing regimens are effective and well-tolerated in Chinese treatment-naïve patients with HIV-1 infection.

## Introduction

Left untreated, human immunodeficiency virus (HIV) infection leads to a severe, life-threatening immunodeficiency syndrome. Worldwide, 36.9 million people were estimated to be living with HIV at the end of 2017 (World Health Organization Global Health Observatory (GHO), 2018; UNAIDS, 2018). The incidence of HIV infection has remained stable since 2005, but the number of people living with HIV (i.e. the prevalence of HIV infection) is steadily increasing (GBD 2015 HIV Collaborators, 2016). HIV prevalence is low in China, but this country is nevertheless ranked seventh worldwide in terms of the total number of infections, with 849,602 HIV-infected individuals registered by the end of September, 2018 (NCAIDS, NCSTD, China CDC, 2018), and this number probably remains underestimated due to inadequate surveillance and under reporting in low-income regions.

Antiretroviral therapy (ART) reduces the risk of disease progression and prevents HIV-1 transmission. Most ART guidelines worldwide recommend its use for all patients with HIV-1 infection, regardless of their CD4 T-cell counts. The China’s National Free Antiretroviral Treatment Program (NFATP) was set up in 2003 and has significantly expanded access to ART in China. Morbidity and mortality in HIV-infected patients have decreased markedly in the last few years (AIDS and Hepatitis C Professional Group, 2018; Liu et al., 2018), but the availability of antiretroviral regimens remains limited in China, as in many developing countries.

According to the NFATP guidelines, the first-line regimen the treatment of HIV infection should consist of a combination of two nucleoside reverse transcriptase inhibitors (NRTIs) and one non-nucleoside reverse transcriptase inhibitor (NNRTI): lamivudine (3TC), zidovudine (AZT) or tenofovir disproxil fumarate (TDF), and efavirenz (EFV) or nevirapine (NVP) or rilpivirine (RPV) (AIDS Professional Group, 2015). The protease inhibitor lopinavir/ritonavir (LPV/r)-based regimen is usually given as the second-line treatment when patients switch from the first-line regimen because of adverse events or drug resistance. Currently, the majority of HIV-1-infected patients are treated with a panel of free limited drugs provided by the Chinese government. No single tablet regimen such as EFV/3TC/TDF was available in China. However, LPV/r can be administered to treatment-naïve patients under some circumstances, such as in situations in which patients have low CD4^+^ T-cell counts, drug resistance is a concern or testing is not available, the patient is female and wishes to have childbearing demand, the patient has a history of mental illness and cannot take EFV or the patient is allergic to EFV.

EFV and LPV/r are no longer recommended as first-line treatments in most developed countries, because of their adverse effects (Gunthard et al., 2014), but they are still widely used in underdeveloped and developing countries (World Health Organization, 2014). In China, 471,140 patients were registered as receiving ART under the NFATP by the end of 2015 (Liu et al., 2018). TDF, AZT, 3TC, EFV, and LPV/r are the most important antiretroviral drugs in clinical use, especially for LPV/r which is the main PI included in the NFATP (AIDS Professional Group, 2015; AIDS and Hepatitis C Professional Group, 2018). However, the previous study has shown that 22% patients displayed EFV concentrations out of the therapeutic range of 1–4 µg/ml in Chinese patients (13.1% < 1 µg/ml, 9.3% > 4 µg/ml) (Meng et al., 2015). These results show poor adherence with EFV in some patients and for others with excess of EFV, adverse events may occur. Thus, more clinical trials of new combinations need to be tested for new first-line ART regimens to improve adherence and tolerance.

Previous studies have suggested that LPV/r performs well in treatment-naïve HIV-1-infected individuals (Cohan et al., 2015; Ghosh et al., 2016; Jespersen et al., 2018), but little is known about the performance of LPV/r-based regimens in treatment-naïve patients in China. The aim of this study was, therefore, to compare the efficacy and adverse effects of LPV/r plus two NRTIs with those of EFV plus two NRTIs as a first-line ART in HIV-1-infected patients from a tertiary care hospital in Beijing, China.

## Materials and Methods

### Study Population

We performed a follow-up study of patients who registered with the NFATP from July 2012 to January 2017 and were followed at the Center for Infectious Diseases of Beijing Youan Hospital, Capital Medical University in Beijing, China. This center is one of the most important HIV/AIDS health care centers participating in the NFATP in China, where more than 8,000 HIV-infected patients on ART are followed regularly.

**Figure 1:**
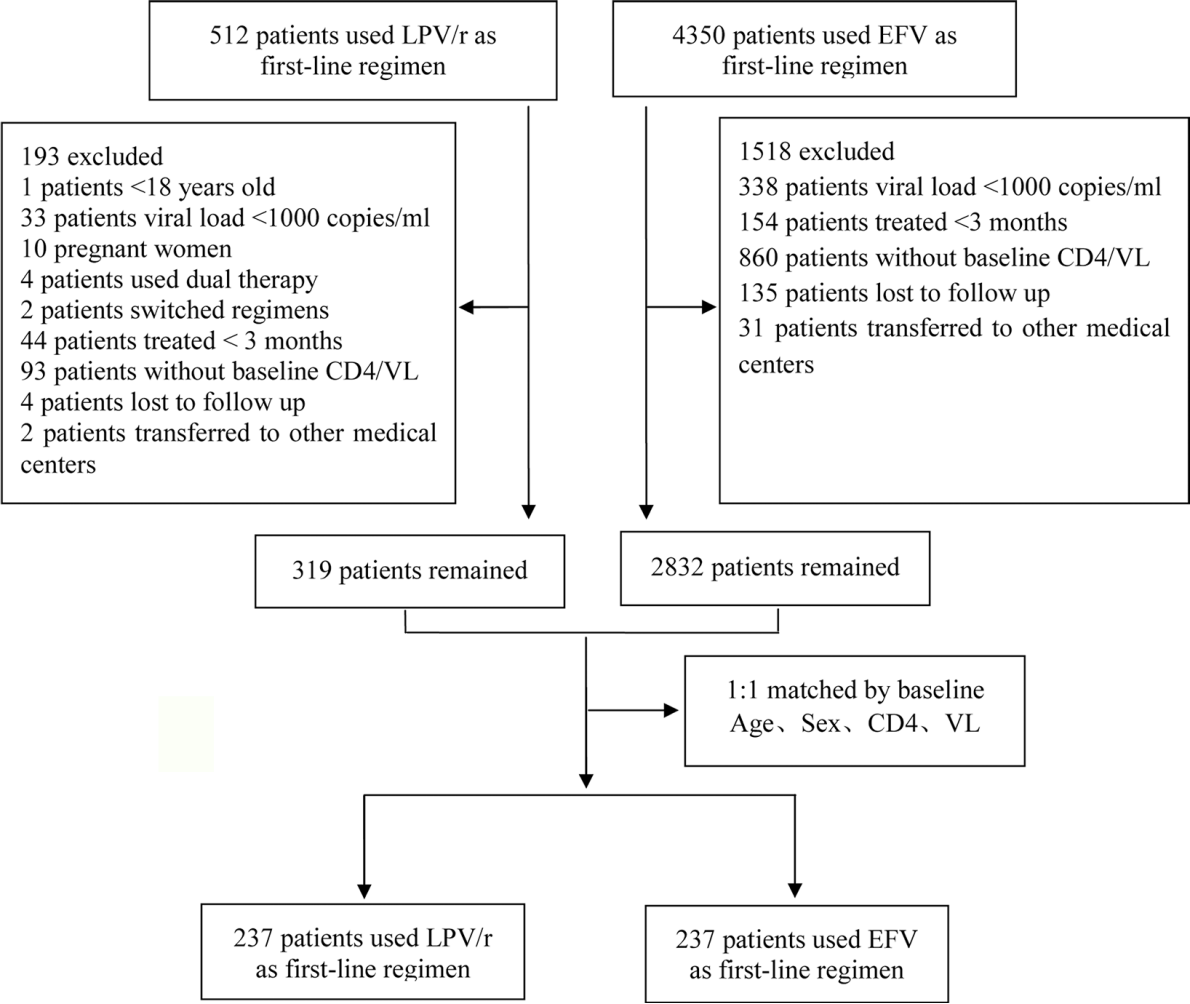
Flowchart for patient selection.

The inclusion criteria were: 1) HIV-1 infection confirmed by western blotting; 2) patient >18 years of age. The exclusion criteria were: 1) pregnancy; 2) use of dual therapy; 3) history of ART before LPV/r therapy; 4) treatment for <3 months; 5) missing baseline data (at least one CD4^+^ T-cell count or plasma HIV viral load at baseline missing); 6) patients who switched regimens. In total, 319 patients were using LPV/r as a first-line regimen and 2,832 patients were using EFV as a first-line regimen. We performed case-control matching to identify the best-matched pairs (1:1). Finally, 237 patients were included in each of the treatment arms in this study (**Figure 1**).

### Ethics Statement

All the participants provided written informed consent for participation in the study and for the storage and use of their clinical samples for research. This study and other related experiments were approved by the Beijing Youan Hospital Research Ethics Committee, and written informed consent was obtained in accordance with the Declaration of Helsinki. The study was carried out in accordance with approved guidelines and regulations.

### Data Collection

Baseline data were collected at treatment initiation. Follow-up visits were scheduled at 2 weeks, 1, 2, and 3 months, and then every three months thereafter. The treatment groups were: 1) LPV/r-based regimen: LPV/r plus TDF/AZT plus 3TC; or 2) EFV-based regimen: EFV plus TDF/AZT plus 3TC. General data (demographic characteristics and symptoms) were collected at baseline. Leukocyte count, hemoglobin level, platelet count, alanine aminotransferase (ALT), total bilirubin, lipid, and glucose levels were determined at baseline and at each visit.

### CD4^+^ T-Cell Count and Viral Load Measurement

CD4^+^ T-cell count and plasma HIV RNA levels were determined at baseline and every six months thereafter. Routine blood CD4^+^ T-cell counts (cells/µl) were measured by four-color flow cytometry with human monoclonal anti-CD4-APC, anti-CD3-FITC, anti-CD8-PE, and anti-CD45-PerCP antibodies (BD Multitest^™^, catalog No. 340499) on peripheral whole-blood samples from each patient according to the manufacturer’s instructions. The cells were analyzed on a BD FACS Canto™ II flow cytometry system (BD Biosciences, San Jose, CA). HIV-1 viral load was determined with an automated real-time PCR-based *m*2000 system (Abbott Molecular Inc, Des Plaines, IL) in accordance with the manufacturer’s instructions with a limit of detection of 40 copies/ml.

### Observation Endpoint

The primary outcome was the proportion of subjects with HIV-1 RNA ≤40 copies/ml at 6, 12, 18 and 24 months. The main secondary endpoint was percentage change in CD4^+^ T-cell count from baseline at 6, 12, 18, and 24 months. Adverse events were assessed by determining: 1) the number of patients who discontinued or switched the ART regimen due to adverse events 2). the number of patients with laboratory abnormalities at least grade 3 due to drugs related. The severity of drug toxicity was evaluated according to the AIDS Clinical Trial Group toxicity grading scale.

### Statistical Analysis

Variables that did not follow a normal distribution are presented as the median and interquartile range (IQR) and were analyzed in Wilcoxon rank sum tests. *P* < 0.05 was used to characterize the statistical significance. Categorical variables as age, sex, CD4^+^ T-cell count, HIV viral load, ART regimens, and laboratory values are presented as numbers and percentages and were analyzed in chi-squared tests. We used linear multilevel models to calculate differences in the change in CD4^+^ T-cell count from baseline to 24 months. Data were managed and analyzed with SAS version 9.14 (SAS Institute, Cary, North Carolina). Differences were considered statistically significant if *P* < 0.05 in two-tailed tests.

## Results

### Characteristics of the Patients

In total, 4,862 patients were included in the study: 237 patients were eligible for analysis in each arm of the study (**Figure 1**). The two groups were comparable at baseline in terms of age, sex, CD4^+^ T-cell count, viral load, and serum lipid concentrations, but LDL-c concentration was higher in the group of patients on the LPV/r-based regimen [2.23 (1.92–2.67) vs. 2.03 (1.92–2.67); *P* < 0.001] (**Table 1**).

**Table 1:** Baseline demographic and clinical characteristics.

Variables	LPV/r-based regimen	EFV-based regimen	*P*-value
	(n = 237)	(n = 237)	
Ages (years)			0.859
< 30	69	64	
30-40	124	126	
≥40	44	47	
Sex			
Men	227 (95.78%)	227 (95.78%)	1.000
Women	10 (4.22%)	10 (4.22%)	
Baseline CD4 (cells/μl)	273.00 (189.00-382.19)	281.50 (161.50-407.50)	0.976
CD4^+^ T-cell count			0.122
≤100	40	27	
> 100 to ≤200	41	43	
> 200 to <350	75	94	
> 350 to <500	45	49	
≥500	36	24	
HIV RNA (log_10_ copies/ml)	4.25 (3.83-4.77)	4.26 (3.83-4.76)	0.942
HIV RNA (log_10_ copies/ml)			1.000
< 100 000	203	203	
≥100 000	34	34	
ART regimen			
TDF+3TC	201	204	0.696
AZT+3TC	36	33	
TC	3.86 (3.42-4.33)	3.89 (3.31-4.36)	0.632
TG	1.06 (0.82-1.47)	1.02 (0.78-1.45)	0.427
HDL-c	0.97 (0.83-1.14)	0.98 (0.85-1.12)	0.847
LDL-c	2.23 (1.92-2.67)	2.03 (1.92-2.67)	<0.001

### Virological Assessment

**Figure 2:**
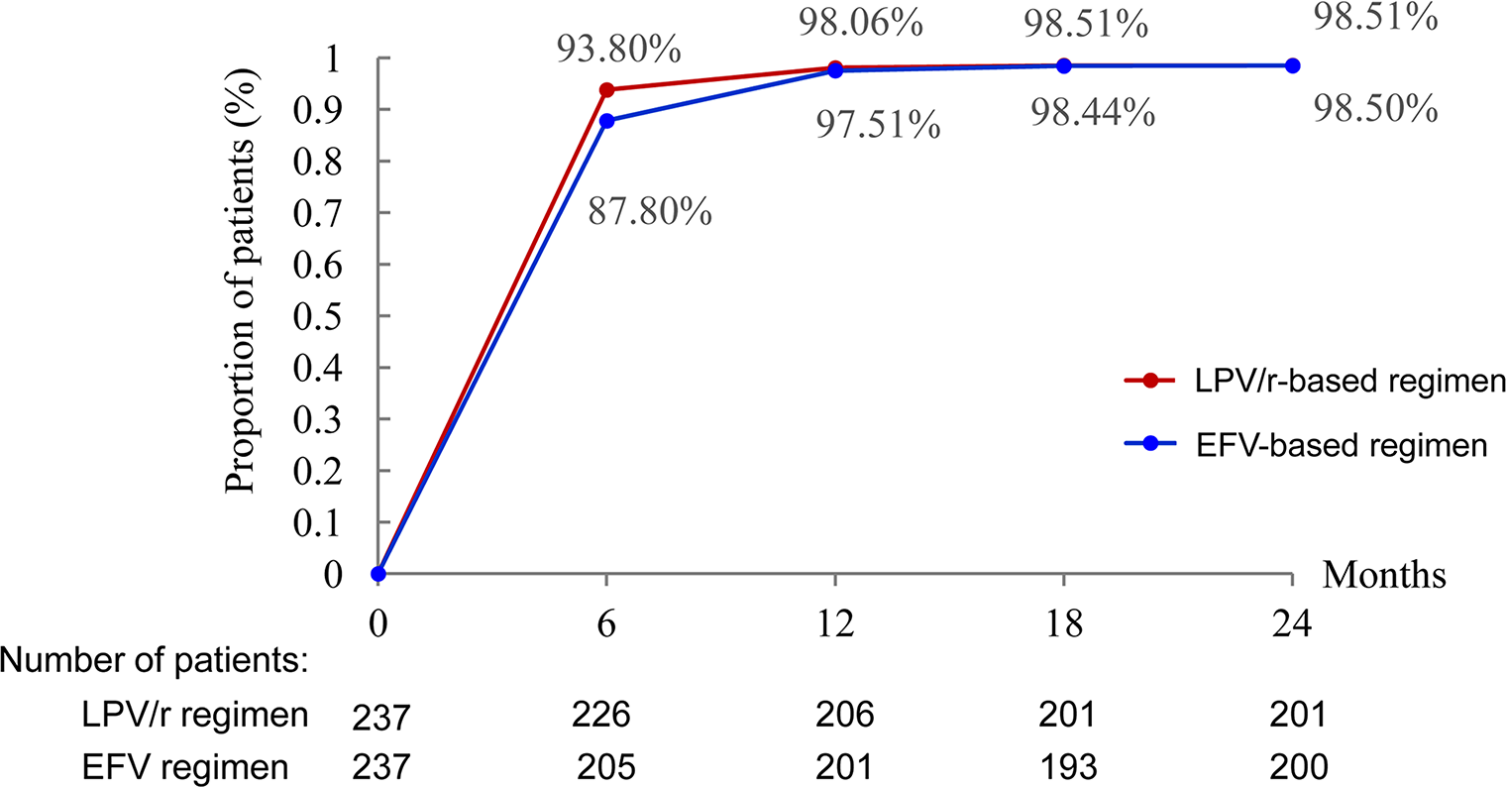
Proportion of patients with HIV RNA <40 copies/ml. *P < 0.05, the difference in the proportion of patients with HIV RNA <40 copies/ml was significant in χ^2^ tests.

During the first six months, virological suppression was better in the LPV/r group than in the EFV group (93.80 vs. 87.80% and *P* < 0.05). Virological suppression rates continued to increase until 12 months, remaining stable thereafter until 24 months in both groups (**Figure 2**).

### Immunological Response

**Figure 3:**
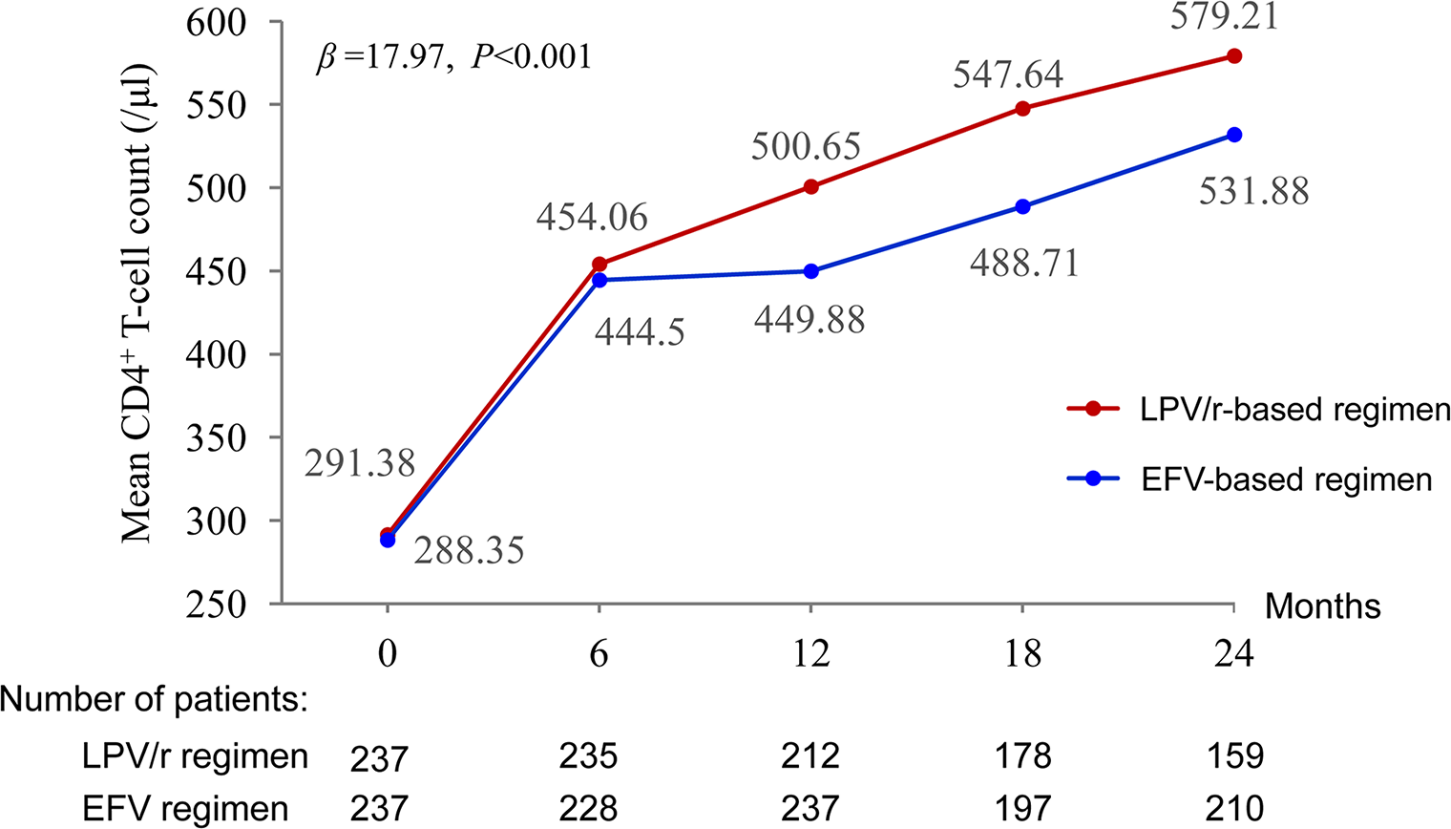
Mean changes in the CD4^+^ T-cell counts of patients.

Mean CD4^+^ T-cell counts increased by 579.21 and 531.88 cells/µl between baseline and 24 months in the LPV/r and EFV groups, respectively. The multilevel analysis revealed that the patients in the LPV/r group were more likely to display an improvement in CD4^+^ T-cell count over time than those in the EFV group (*P* < 0.001) (**Figure 3**).

### Adverse Effects

None of the patients discontinued treatment due to adverse events. Adverse laboratory events of grade 3 or 4 were noted in 14 patients (5.91%) in the LPV/r group and three patients (1.20%) in the EFV group (**Table 2**).

**Table 2:** Laboratory abnormalities at 6, 12, 18, 24 months.

	LPV/r-based regimen	EFV-based regimen
	(n = 237)	(n = 237)
Grade 3 or 4 laboratory abnormalities		
Leukocytes	0	0
Hemoglobin	0	0
Platelets count	0	0
Alanine aminotransferase (ALT)	0	0
Fasting glucose	2	0
Creatinine (CR)	0	0
Total cholesterol	2	1
Triglycerides	9	2
HDL cholesterol	0	0
LDL cholesterol	1	0

## Discussion

Few data are available for the performance of first-line LPV/r-based regimens in treatment-naïve patients with HIV-1 infection (Cohan et al., 2015; Jespersen et al., 2018). This study therefore aimed to assess the efficacy and adverse effects of LPV/r plus 3TC and TDF, or AZT as a first-line antiretroviral therapy in HIV-1-infected individuals, by comparison with a standard EFV-based regimen. The results obtained suggest that LPV/r- based ART has a good efficacy and adverse event profile for the Chinese treatment naïve patients with HIV-1 infection.

ART greatly improves the prognosis of HIV-infected patients, but factors such as adverse drug reactions, inadequate compliance, and drug resistance increase the likelihood of clinical and virological failure (Ghosn et al., 2018; Prabhu et al., 2019). LPV/r still plays a key role in treatment in developing countries, despite being an old drug that is no longer recommended for first-line treatment in Western countries, in which it is more widely used as a second-line therapy (Developed by the DHHS Panel on Antiretroviral Guidelines for Adults and Adolescents, 2019). In areas with limited resources, such as China, LPV/r-based regimens are free and are the only option in situations in which EFV cannot be used due to primary drug resistance, allergies, hepatotoxicity, neurotoxicity, or pregnancy plans, for example. It thus remains a key drug in China’s current anti-HIV treatment program.

Patients who agreed to ART with a LPV/r-based regimens displayed high rates of virological suppression and good immunological recovery. A study in pregnant women showed that first-line EFV- and LPV/r-based regimens both led to high levels of virological suppression and a low risk of transmission to the infant (Cohan et al., 2015). An African study showed that first-line LPV/r-based regimens triggered lower rates of treatment resistance than NNRTIs, but were not superior in terms of efficacy or severe adverse events (Jespersen et al., 2018). However, six studies have described patients who received EFV-containing regimens presented poor adherence due to neuropsychiatric adverse such as body heat, delusions, dizziness, anxiety, intense, and nightmares (Li et al., 2017), which can be associated with NNRTI resistance.

In our patients, virological suppression was achieved within the first six months in 93.8% of the patients who received initial LPV/r-based regimens. Viral suppression took longer to achieve in patients with baseline viral loads >100,000 copies/ml, consistent with the findings of previous study (Haile et al., 2016). Regardless of the stratification method used, the initial treatment of patients with LPV/r-based regimens resulted in complete virological suppression within 18 months. Some previous studies using a cutoff value of 40 copies/ml to define virological suppression have reported the achievement of virological suppression in 70% of patients on LPV/r-based regimens (Antiretroviral Therapy Cohort Collaboration, 2017).

In addition, another randomized controlled trial (ACTG) in Africa and Asia showed that >80% of the patients had suppressed plasma HIV RNA levels from week 12 onward (< 400 copies/ml) when treated with the LPV/r monotherapy (Kumarasamy et al., 2015). The greater benefits of a rapid decrease in plasma HIV RNA levels, such as the prevention of HIV infection in HIV-negative individuals at high risk of exposure have been demonstrated in increasing numbers of studies. The prevalence of resistance to NNRTIs among previously untreated individuals living in areas of limited resources, such as Sub-Saharan Africa (5% resistance rate) (Gupta et al., 2012) and South Korea (2.7% resistance) (Park et al., 2016), has increased, and PI-based regimens may be more appropriate in these areas. The widespread use PI-based first-line therapies in resource-limited settings is not currently recommended, due to the high risk of resistance-related failure for second-line NNRTI/NRTI regimens (Hill et al., 2013), but Hill *et al*. suggested that LPV/r-based regimens might be superior to NNRTIs as an initial treatment, particularly in limited-resource settings, in which there may be resistance, but no access to drug resistance testing (Hill et al., 2013).

In this study, mean CD4^+^ T-cell count had increased by 209.3 and 287.8 cells/µl relative to baseline at 12 and 24 months, respectively. Baseline CD4^+^ T-cell counts are associated with immune response after ART. Patients with a CD4^+^ T-cell count <200 cells/µl at the start of treatment had poorer immunologic outcomes than patients with >200 cells/µl, consistent with the findings of previous studies (Garcia et al., 2004). In this study, most patients had high CD4^+^ T-cell counts at baseline, higher than those for patients on second-line ART in other studies (Luz et al., 2015), but similar to those reported in another study assessing LPV/r-based second-line ART (Patel et al., 2013). In an Iranian study, the authors reported that mean CD4^+^ T-cell counts had increased by 139 cells/µl relative to baseline at 12 months (Rasooli-Nejad et al., 2017). The authors of a Ugandan study reported an increase in mean CD4^+^ T-cell counts of 153 cells/µl at 12 months (Laker et al., 2014). We therefore hypothesized that initial treatment with LPV/r-based regimens might lead to a better immune response than switching to LPV/r-based regimens after first-line ART failure. Further studies are required to test this hypothesis.

Some adverse events were observed in our study population, but tolerance was good in most patients, and none of the patients discontinued treatment due to adverse events. An increase in LDL-c levels is one of the most important risk factors for atherosclerotic cardiovascular disease (ASCVD) (Stone et al., 2014a; Stone et al., 2014b; Catapano et al., 2017). Reducing LDL-c levels also reduces the risk of ASCVD and death (Baigent et al., 2005). We show here that median LDL-c levels had not increased after 24 months of ART. Similarly, HDL-c and TC levels remained good after 24 months (**Table 1**). In other studies, LPV/r-based regimens have generally been reported to be well-tolerated in terms of changes in lipid levels (Molina et al., 2007; Gathe et al., 2009), suggesting a limited impact of LPV/r-based regimens on cardiovascular risk. The results of this study suggest that the lipid profiles of patients taking AZT were generally poorer than those of patients taking TDF, especially for TC and LDL-c, but these differences were not significant, due to the small number of cases. A similar trend has been reported in previous studies (Feeney and Mallon, 2011; Souza et al., 2013; da Cunha et al., 2015; Ombeni and Kamuhabwa, 2016). No serious adverse events or severe hepatic dysfunction associated with LPV/r-based ART were observed. No patient discontinued ART or switched regimens because of adverse events.

However, some limitations of this study deserve mention. The sample size was small and all the patients came from a single center, limiting the extent to which the results can be generalized in whole China. No control group receiving a NNRTI was included. In addition, for reasons that were not always recorded on the patients’ medical charts, only 159 patients were still in follow-up at 24 months, corresponding to an attrition rate of 33%. In addition, the higher loss of follow-up in the group of LPV/r may be related to the bi-daily administration, and/or to the digestive tract side effects. The data were limited to those available from the medical charts, and it was not possible to test additional biomarkers or factors associated with treatment failure. Additional studies are required to address these issues.

In conclusion, LPV/r-based ART was found to be beneficial and well-tolerated as a first-line ART in a resource-limited setting such as China. We observed high rates of virological suppression, immunological responses, and tolerability in treatment-naïve patients. Long-term (24 months) treatments with TDF+3TC+LPV/r or AZT+3TC+LPV/r were similarly beneficial. The use of LPV/r-based ART for treatment-naïve patients could be beneficial for patients for whom drug resistance testing is not available.

## Data Availability Statement

All datasets generated for this study are included in the article/supplementary material.

## Ethics Statement

The studies involving human participants were reviewed and approved by the Beijing Youan Hospital Research Ethics Committee, Beijing Youan Hospital, Capital Medical University. The patients/participants provided their written informed consent to participate in this study.

## Author Contributions

BS, YW, LD, and LS conceived the study, designed the experiments, and analyzed the data. TJ, HZ, ZL, AL, YS, and WH performed the experiments, carried out the data collection and data analysis. RZ, TZ, HW, SH, and LS contributed to reagents and materials. BS, YW, and LD wrote the article and revised the manuscript. All authors read and approved the final manuscript.

## Funding

This work was supported by the National 13^th^ Five-Year Grand Program on Key Infectious Disease Control (2017ZX10202102-005-003 to BS, 2018ZX10721102-003-003 and 2018ZX10302-102 to LD, 2018ZX10301-407-005 and 2018ZX10302103-001-003 to TJ, 2017ZX10202101-004-001 to TZ), the National Natural Science Foundation of China (NSFC, 81772165 to BS, 81571973 to HW, 81601795 to LD), the Beijing Municipal of Science and Technology Major Project (D161100000416003 to HW), Beijing Municipal Administration of Hospitals’ Youth Program (QML20161702), and the Beijing Key Laboratory for HIV/AIDS Research (BZ0089). The funders had no role in study design, data collection and analysis, decision to publish, or preparation of the manuscript.

## Conflict of Interest

The authors declare that the research was conducted in the absence of any commercial or financial relationships that could be construed as a potential conflict of interest.
